# Correlations between physical activity and quality of life in entrepreneurs from Wrocław, Poland

**DOI:** 10.1186/s13102-023-00624-4

**Published:** 2023-02-02

**Authors:** Daniel Puciato, Dawid Bączkowicz, Michał Rozpara

**Affiliations:** 1 Faculty of Physical Education and Sport, Wroclaw University of Health and Sport Sciences, al. Ignacego Jana Paderewskiego 35, 51-612 Wrocław, Poland; 2 Faculty of Physical Education and Physiotherapy, Opole University of Technology, Prószkowska 76, 45-578 Opole, Poland; 3 Institute of Sport Sciences, The Jerzy Kukuczka Academy of Physical Education, Mikołowska 72A, 40-065 Katowice, Poland

**Keywords:** Physical activity, Quality of life, Entrepreneurs, Poland

## Abstract

**Background:**

Limited physical activity is one of the main reasons for the rapid increase in the prevalence of diseases of affluence, which can lead to premature deaths in adults. Quality of life may be one of potential determinants of physical activity. The aim of this article is to identify the relationships between physical activity and quality of life in entrepreneurs from Wroclaw, Poland.

**Methods:**

A cross-sectional study was conducted on a group of 616 entrepreneurs (216 women and 400 men). A diagnostic survey method was used with a direct interview technique. The research tools included the International Physical Activity Questionnaire Short Form and the World Health Organization Quality of Life Questionnaire.

**Results:**

Among the studied entrepreneurs, along with their higher ratings of overall quality of life, the odds of high rather than low levels of physical activity increased nearly fivefold (OR 4.86, CI 3.34–7.07). Entrepreneurs with higher assessment levels of their perceived health condition were nearly twice as likely to report high rather than low physical activity levels (OR 1.92, CI 1.42–2.59). The conditional probability of high rather than low levels of physical activity also increased in the entrepreneurs with higher assessments of quality of life in the physical, psychological, social, and environmental domains, by 34%, 11%, 5%, and 6%, respectively.

**Conclusion:**

Programs promoting physical activity among entrepreneurs, which focus on moderate and high intensity exercise, should be considered desirable in the context of the study findings. Activities aimed at improving quality of life in the physical and psychological domains as a potential determinant of the level of physical activity of entrepreneurs are also worth recommending.

## Background

Reduced physical activity is one of the main reasons for the rapid increase in the prevalence of diseases of affluence, which can lead to premature deaths in adults [1, 2]. Sedentary lifestyle and low physical activity levels have a number of negative consequences for the musculoskeletal [3], circulatory [4], respiratory [5], digestive [6], nervous [7], and endocrine [8] systems.

Physical activity (PA) has also a positive impact on psychological well-being in the cognitive, emotional, and volitional spheres [9, 10]. It is particularly important for entrepreneurs, who are often heavily burdened with work and exposed to excessive stress [11] contributing to occupational burnout [12], and consequently, to the deterioration of some quality of life indicators [13, 14]. This was confirmed by Charles-Leija et al. [15], who showed that entrepreneurs were characterized by lower levels of satisfaction with life and health than their employees. It is also noteworthy that regular PA may indirectly benefit some entrepreneurial qualities such as autonomous motivation [16, 17], self-efficacy [18, 19], positive social activity [20, 21], and high self-assessment [22]. Lundberg and Fredman [23] also demonstrated that entrepreneurs’ lifestyle, including physical activity, can be associated with business success.

Despite the significance of the subject of this paper, there have been very few studies to date regarding the physical activity of entrepreneurs. Gu et al. [24] demonstrated that 56.2% of the surveyed entrepreneurs met health-related standards of leisure-time physical activity. On the other hand, Dodson et al. [25] proved that individuals in business occupations were more likely to engage in leisure time physical activity when it involved attractive challenges and high-level physical efforts. Furthermore, the entrepreneurs were more likely to meet PA recommendations through engaging in commuting physical activities when they had access to worksite showers and incentives to bike or walk.

Determinants of physical activity are an important and frequently addressed research issue. According to an ecological model proposed by Sallis et al. [26], human physical activity is influenced by intrapersonal, interpersonal, and environmental determinants in regional, national and global terms. The first group of PA determinants includes biological factors (e.g. genes, health status) and psychological factors (e.g. motivation, cognitive processes, values, emotions). The second group of factors comprises the effects on individuals engaged in physical activity, their family, friends, colleagues, as well as cultural norms and social practices. The environmental factors can be natural (e.g. climate, topography), urban (e.g. land use, safety, transport, or availability of recreational services in the place of residence and work), and social (e.g. physical activity behaviours of other people, or organisational practices in the workplace). Regional and national factors include health care, education system, and attitudes towards physical activity. Global factors are mainly economic development, socio-cultural norms, and physical activity trends [26].

The concept of quality of life and such conceptual categories as [27] living standards, well-being, life satisfaction, human flourishing, life happiness, and life dignity correspond well with the ecological model. Quality of life is today one of the fundamental categories in multiple scholarly disciplines and in socio-economic practice. There are several strands of quality of life in literature, which can roughly be divided into those focusing on [28]: environmental protection (effects of civilization progress on the environment, human health and quality of life, e.g., air and water pollution); urbanization (consequences of science and technology development in urbanized areas, e.g. excessive number of cars); socio-psychological issues (effects of civilization progress on individuals and social life, e.g., loneliness or alienation); and economic issues (quality of life understood as the standard of living of an individual, local community, region, country, or international communities, e.g., wealth or poverty levels or the Human Development Index) as well as health status (effects of health condition on quality of life, e.g. assessment of physical or psychological functioning).

In the view of authors of this study, the measurement of health-related quality of life (HRQoL) with regard to entrepreneurs deserves special attention for several reasons. First, as already emphasized, entrepreneurs’ physical activity levels are mostly not satisfactory. Secondly, entrepreneurs are exposed to a number of adverse effects on their health status and quality of life, i.e.: limited free time resources, high levels of stress, or heavy workload, all of which are signifcant to their health status. Thirdly, entrepreneurs are crucial for the socio-economic development of their countries or regions. Fourth, all other concepts of quality of life emphasize the vital role of health status.

The World Health Organization defines quality of life as “an individual’s perception of their position in life in the context of the culture and value systems in which they live and in relation to their goals, expectations, standards and concerns” [29]. The most commonly implemented research tool within this concept is the World Health Organization Quality of Life—WHOQOL BREF questionnaire, also used in the present study, which considers areas such as overall quality of life, life satisfaction and health-related quality of life in the physical, psychological, social, and environmental domains [29].

In previous studies, however, the relationships between quality of life and physical activity in people of working age were considered quite frequently [30–35]. These relationships were particularly evident for quality of life in the physical and psychological domains [36, 37]. The conducted literature review indicates, therefore, two clear research gaps. Firstly, the relationships between physical activity and quality of life in people of working age have not been considered before. Secondly, entrepreneurs have rarely been the subject of earlier studies.

The aim of this study was to examine the relationships between physical activity and the quality of life of entrepreneurs from Wrocław, Poland. The following research hypotheses were formulated:

### **Hypothesis 1**

The dominant proportion of the surveyed entrepreneurs is characterized by a high level of physical activity.

### **Hypothesis 2**

The level of physical activity of the entrepreneurs is positively correlated with their perceived health condition.

### **Hypothesis 3**

Among the entrepreneurs, there are positive associations of the level of physical activity with the assessment of overall quality of life and quality of life in the physical, psychological, social, and environmental domains.

## Methods

The interviews were conducted in the city of Wrocław in southwestern Poland, located near the border with the Czech Republic and Germany, with a population of about 638,000. Wrocław is the fastest growing Polish city and has repeatedly been ranked among the top cities in the European Union in terms of the rate of socio-economic development. Wrocław also occupies a high, third place in Poland, in terms of number of business entities per 10 thousand inhabitants. Research results also show that Wrocław has some of the highest quality of life indicators in Poland [38]. The above mentioned premises were the most important reasons for selecting Wrocław as the study location.

The study was conducted between 2014 and 2016 on a group of 616 entrepreneurs. An entrepreneur is defined as a natural person, a legal person, or an organizational unit that is not a legal person but is granted legal capacity by law and conducts business activities. Entrepreneurs are also partners in civil law partnerships within the scope of their business activity. The entrepreneurs were singled out from the group of 4332 respondents (2276 women and 2056 men) who had participated in research on the socio-economic situation, quality of life, and physical activity of working-age residents of Wrocław.

The sample size was estimated according to the following formula for finite population [39]:

$$n = \frac{{Nz^{2} p(1 - p)}}{{E^{2} (N - 1) + z^{2} p(1 - p)}}$$

where *N*—number of Wrocław inhabitants as of December 31, 2013 (*N* = 632,067); *p*—proportion of Wrocław working age population as of December 31, 2013 (*p* = 0.63); *E*—margin of error (*E* = 0.015); *z*—z-score associated with a 95% confidence interval (*z* = 1.96).

The sample size was increased by 15% from the original estimate to account for potential refusals to participate in the survey.

The sample selection was of multistage and mixed character (random and purposive). In the first stage, using a random number table, 10 housing estates in Wrocław were drawn. In the second stage, using the same random mechanism, 3 streets were selected from each of the 10 housing estates. In the last stage, from among passers-by encountered in the selected streets, every fourth person was asked to participate in the survey. The following inclusion criteria were assumed: address of residence in one of the selected streets and working age (18–64 years). The exclusion criteria involved chronic diseases, e.g., cancer, diabetes, arterial hypertension, osteoarthritis, osteoporosis. All respondents were informed about the purpose and course of the survey and their voluntary participation and asked to provide their informed consent to participate. Out of 4595 individuals who were asked to participate in the study, 6% (262) declined to take part in the survey.

The study was cross sectional. The diagnostic survey method was employed using the direct interview technique.

The research tool used to measure respondents' physical activity was the International Physical Activity Questionnaire Short Form (IPAQ-SF) [40], consisting of 6 items concerning the frequency (F) and time (T) of physical activity at three intensity levels: vigorous (VPA), moderate (MPA), and light (LPA).

Based on the responses to the questionnaire items, the physical activity level (PAL) of entrepreneurs was assessed as the dependent variable (DV).

The following PAL categories were identified [41]:

1. High physical activity level (HPAL): respondents meeting at least one of the two criteria:
  - 3 or more days of vigorous-intensity exercise with a total energy expenditure of at least 1500 MET min/week,
  - 7 days or more of any combination of physical activity (of light, moderate and vigorous intensity) with a total energy expenditure of at least 3000 MET min/week.
2. Moderate physical activity level (MPAL): respondents meeting one of three criteria:
  - 3 or more days of vigorous-intensity exercise lasting no less than 20 min per day,
  - 5 or more days of moderate or light intensity exercise for no less than 30 min per day,
  - 5 days or more of any combination of light, moderate, or vigorous-intensity physical activity.
3. Low physical activity level (LPAL) respondents not displaying any physical activity or not meeting the requirements for moderate or vigorous levels of physical activity.

Respondents’ quality of life was assessed using the World Health Organization Quality of Life (WHOQOL-BREF) questionnaire [29]. The questionnaire consists of 26 items evaluating overall quality of life (OQoL), perceived health condition (PHC), and quality of life in four domains: physical (PHYD), psychological (PSYD), social (SD) and environmental (ED) as independent variables (IVs).

During the interviews data were also obtained on respondents' gender (female, male), age (up to 44 years, 44 years and more), education (primary, basic vocational, secondary, higher), and marital status (single, married) as confounding variables (CVs).

The descriptive statistics of frequency (f) and relative frequency (rf), mean (M), median (Me), standard deviation (SD) and quartile deviation (QD) were calculated for the quality of life, perceived health condition and physical activity indices. Multinomial logistic regression (MRL) was used to assess the associations between physical activity (DV) and quality of life and perceived health condition (IV) in the variant of sequential introduction of variables into the model. Raw and adjusted for confounding variables odds ratio (OR), likelihood ratio (LR) and accuracy of prediction (ACC) were determined. Confidence interval (95%CI) for rf, M, Me, and OR were also reported. The level of statistical significance was set at α = 0.05. All statistical calculations were made using IBM SPSS Statistics 26 (IBM Corporation, Armonk, NY, USA).

## Results

Table 1 shows the socio-demographic characteristics of Wrocław entrepreneurs. 65% of respondents were men, and 35% were women. About 64% of the respondents were aged up to 44 years, while 36% were over 44 years. Half of the respondents had a higher education, 31% secondary education, and 18% primary or basic vocational education. Almost 83% were married, and about 17% were single.

**Table 1:** Socio-demographic characteristics of Wrocław entrepreneurs

Variable	*f*	*rf* (95% CI for *rf*)
*Gender*		
Female	216	35.1 (31.3–38.8)
Male	400	64.9 (61.2–68.7)
*Age*		
Below 44 years	396	64.3 (60.5–68.1)
Above 44 years	220	35.7 (31.9–39.5)
*Education*		
Primary	114	18.5 (15.4–21.6)
Secondary	192	31.2 (27.5–34.8)
Higher	310	50.3 (46.4–54.3)
*Marital status*		
Single	107	17.4 (14.4–20.4)
Married	509	82.6 (61.2–68.7)

*f* frequency, *rf* relative frequency (percentage)

The caloric cost of respondents' total physical activity ranged from 2559.0 ± 1636.5 METmin/week. The energy expenditure of vigorous-intensity physical activity was 1440.0 ± 960.0 METmin/week, moderate-intensity—960.0 ± 480.0 METmin/week, and light-intensity—693.0 ± 528.0 METmin/week. The entrepreneurs were most likely to engage in light-intensity physical activity (5.0 ± 1.0 day/week), and least likely to engage in moderate- and light-intensity physical activity (3.0 ± 1.0 day/week each). The duration of light and vigorous intensity physical activity was 60.0 ± 15.0 min/day each, and moderate-intensity physical activity was 60.0 ± 30.0 min/day (Table 2).

**Table 2:** Physical activity and quality of life of Wrocław entrepreneurs

Variable	*M* (95% CI for *M*)	*SD*	*Me* (95% CI for *Me*)	*QD*
*Physical activity*				
FVPA (day/week)	3.1 (3.0–3.3)	1.5	3.0 (3.0–4.0)	1.0
TVPA (min/day)	73.7 (69.6–77.8)	40.3	60.0 (60.0–90.0)	15.0
EEVPA (METmin/week)	1930.9 (1780.6–2081.2)	1478.5	1440.0 (1440.0–1920.0)	960.0
FMPA (day/week)	3.4 (3.3–3.6)	1.4	3.0 (3.0–4.0)	1.0
TMPA (min/day)	85.7 (80.8–90.7)	50.7	60.0 (60.0–90.0)	30.0
EEMPA (METmin/week)	1208.6 (1113.3–1303.9)	971.9	960.0 (960.0–1260.0)	480.0
FLPA (day/week)	4.7 (4.6–4.8)	1.6	5.0 (5.0–7.0)	1.0
TLPA (min/day)	61.5 (57.7–65.2)	46.0	60.0 (60.0–90.0)	15.0
EELPA (METmin/week)	1026.8 (944.9–1108.6)	1008.2	693.0 (693.0–792.0)	528.0
EEPA (METmin/week)	2936.1 (2737.5–3134.7)	2510.0	2559.0 (2175.0–2820.0)	1636.5
*Quality of life and perceived health condition*				
OQoL (pts.)	3.8 (3.8–3.9)	0.7	4.0 (4.0–5.0)	0.5
PHC (pts.)	3.7 (3.6–3.7)	0.8	4.0 (4.0–5.0)	0.5
PHYD (pts.)	12.2 (12.0–12.3)	1.6	12.0 (12.0–13.0)	0.5
PSYD (pts.)	13.7 (13.6–13.9)	1.7	14.0 (14.0–15.0)	0.5
SD (pts.)	15.0 (14.8–15.2)	2.3	15.0 (15.0–16.0)	1.0
ED (pts.)	13.6 (13.4–13.8)	2.4	13.0 (13.0–14.0)	1.0

*FVPA* frequency of vigorous-intensity physical activity, *TVPA* time of vigorous-intensity physical activity, *EEVPA* energy expenditure of vigorous-intensity physical activity, *FMPA* frequency of moderate-intensity physical activity, *TMPA* time of moderate-intensity physical activity, *EEMPA* energy expenditure of moderate-intensity physical activity, *FLPA* frequency of light-intensity physical activity, *TLPA* time of light-intensity physical activity, *EELPA* energy expenditure of light-intensity physical activity, *EEPA* energy expenditure of total physical activity, *OQoL* overall quality of life, *PHC* perceived health condition, *PHYD* physical domain of quality of life, *PSYH* psychological domain of quality of life, *SD* social domain of quality of life, *ED* environmental domain of quality of life, *Me* mean, *SD* standard deviation, *Me* median, *QD* quartile deviation

The assessment of the overall quality of life and perceived health condition of Wrocław entrepreneurs was within 3.8 ± 0.7 pts. Respondents rated their quality of life in the social (15.0 ± 2.3 pts.) and psychological (13.7 ± 1.7 pts.) domains higher than in the physical (12.2 ± 1.6 pts.) and environmental (13.6 ± 2.4 pts.) domains (Table 2).

According to the physical activity assessment criteria from the Guidelines for Data Processing and Analysis of the International Physical Activity Questionnaire [37] the Wrocław entrepreneurs were predominantly (46%) highly physically active. Only 27% of the entrepreneurs reported moderate and low physical activity levels (Table 3).

**Table 3:** Physical activity levels of Wrocław entrepreneurs

Variable	*f*	*rf* (95% CI for *rf*)
*Physical activity level*		
HPAL	283	45.9 (42.0–49.9)
MPAL	169	27.4 (23.9–31.0)
LPAL	164	26.6 (23.1–30.1)

*HPAL* high level of physical activity, *MPAL* moderate level of physical activity, *LPAL* low level of physical activity, *f* frequency, *rf* relative frequency (percentage)

Table 4 presents the results of multinomial logistic regression analysis representing raw and CVs-adjusted associations of physical activity (DV) with overall quality of life, perceived health condition, and quality of life in the physical, psychological, social, and environmental domains (IVs) of the entrepreneurs from Wrocław.

**Table 4:** Relationships between physical activity levels and quality of life of private entrepreneurs from Wroclaw

PAL^a^	Predictor	Model M_1_		Model M_2_		Model M_3_		Model M_4_		Model M_5_	
		OR^b^ (95% CI for OR)	ACC	OR^c^ (95% CI for OR)	ACC	OR^d^ (95% CI for OR)	ACC	OR^e^ (95% CI for OR)	ACC	OR^f^ (95% CI for OR)	ACC
Model fitting		LR_M1_ (*χ*^*2*^ = 74.6, *df* = 2, *p* < .001)		LR_M1–M2_ (χ^2^ = 32.5, *df* = 2, *p* < .001)		LR_M2–M3_ (χ^2^ = 0.9, *df* = 2, *p* = .651)		LR_M3–M4_ (χ^2^ = 120.6, *df* = 4, *p* < .001)		LR_M4–M5_ (χ^2^ = 30.3, *df* = 2, *p* < .001)	
HPAL	OQoL (pts.)	3.16 (2.32–4.28)	49.5%	3.12 (2.28–4.26)	50.3%	3.09 (2.26–4.24)	50.3%	4.32 (3.01–6.19)	60.1%	4.86 (3.34–7.07)	61.9%
MPAL		1.28 (0.93–1.76)		1.26 (0.90–1.74)		1.26 (0.91–1.76)		1.29 (0.92–1.81)		1.27 (0.90–1.78)	
Model fitting		LR_M1_ (χ^2^ = 32.1, *df* = 2, *p* < .001)		LR_M1–M2_ (χ^2^ = 29.6, *df* = 2, *p* < .001)		LR_M2–M3_ (χ^2^ = 11.1, *df* = 2, *p* = .004)		LR_M3–M4_ (χ^2^ = 83.4, *df* = 4, *p* < .001)		LR_M4–M5_ (χ^2^ = 24.0, *df* = 2, *p* < .001)	
HPAL	PHC (pts.)	2.04 (1.57–2.63)	51.3%	1.89 (1.46–2.46)	48.2%	2.13 (1.62–2.81)	51.1%	1.87 (1.39–2.53)	58.0%	1.92 (1.42–2.59)	63.1%
MPAL		1.32 (1.02–1.72)		1.18 (0.90–1.55)		1.20 (0.90–1.60)		1.07 (0.80–1.42)		1.03 (0.77–1.38)	
Model fitting		LR_M1_ (χ^2^ = 38.7, *df* = 2, *p* < .001)		LR_M1–M2_ (χ^2^ = 40.9, *df* = 2, *p* < .001)		LR_M2–M3_ (χ^2^ = 13.0, *df* = 2, *p* = .002)		LR_M3–M4_ (χ^2^ = 96.2, *df* = 4, *p* < .001)		LR_M4–M5_ (χ^2^ = 22.7, *df* = 2, *p* < .001)	
HPAL	PHYD (pts.)	1.50 (1.31–1.73)	49.0%	1.56 (1.36–1.80)	50.5%	1.70 (1.46–1.98)	51.9%	1.80 (1.52–2.14)	53.2%	1.84 (1.54–2.19)	55.4%
MPAL		1.37 (1.18–1.58)		1.43 (1.23–1.67)		1.52 (1.29–1.79)		1.56 (1.31–1.86)		1.54 (1.29–1.84)	
Model fitting		LR_M1_ (χ^2^ = 23.5, *df* = 2, *p* < .001)		LR_M1–M2_ (χ^2^ = 34.3, *df* = 2, *p* < .001)		LR_M2–M3_ (χ^2^ = 5.1, *df* = 2, *p* = .078)		LR_M3–M4_ (χ^2^ = 100.9, *df* = 4, *p* < .001)		LR_M4–M5_ (χ^2^ = 22.9, *df* = 2, *p* < .001)	
HPAL	PSYD (pts.)	1.20 (1.07–1.36)	48.1%	1.24 (1.09–1.40)	47.7%	1.25 (1.10–1.42)	45.5%	1.28 (1.12–1.47)	58.8%	1.31 (1.14–1.51)	58.0%
MPAL		0.91 (0.81–1.03)		0.95 (0.84–1.09)		0.96 (0.84–1.09)		0.93 (0.81–1.07)		0.94 (0.81–1.08)	
Model fitting		LR_M1_ (χ^2^ = 42.7, *df* = 2, *p* < .001)		LR_M1–M2_ (χ^2^ = 34.3, *df* = 2, *p* < .001)		LR_M2–M3_ (χ^2^ = 6.2, *df* = 2, *p* = .045)		LR_M3–M4_ (χ^2^ = 94.4, *df* = 4, *p* < .001)		LR_M4–M5_ (χ^2^ = 23.1, *df* = 2, *p* < .001)	
HPAL	SD (pts.)	1.23 (1.11–1.36)	49.4%	1.22 (1.01–1.35)	49.8%	1.24 (1.11–1.37)	47.6%	1.24 (1.11–1.39)	61.2%	1.28 (1.14–1.44)	58.3%
MPAL		0.91 (0.83–0.99)		0.90 (0.81–0.99)		0.90 (0.82–0.99)		0.89 (0.81–0.99)		0.90 (0.81–1.00)	
Model fitting		LR_M1_ (χ^2^ = 49.1, *df* = 2, *p* < .001)		LR_M1–M2_ (χ^2^ = 38.2, *df* = 2, *p* < .001)		LR_M2–M3_ (χ^2^ = 2.6, *df* = 2, *p* = .268)		LR_M3–M4_ (χ^2^ = 97.6, *df* = 4, *p* < .001)		LR_M4–M5_ (χ^2^ = 19.0, *df* = 2, *p* < .001)	
HPAL	ED (pts.)	1.27 (1.16–1.40)	44.5%	1.32 (1.20–1.45)	48.7%	1.32 (1.20–1.45)	49.7%	1.32 (1.19–1.47)	56.2%	1.33 (1.20–1.49)	59.4%
MPAL		0.96 (0.88–1.05)		1.01 (0.92–1.11)		1.01 (0.92–1.11)		0.96 (0.87–1.05)		0.97 (0.88–1.07)	

*HPAL* high physical activity level, *MPAL* moderate physical activity level, *OQoL* overall quality of life, *PHC* perceived health condition, *PHYD* physical domain of quality of life, *PSYD* psychological domain of quality of life, *SD* social domain of quality of life, *ED* environmental domain of quality of life

^a^The reference category is LPAL

^b^Unadjusted odds ratio

^c^Adjusted odds ratio for gender

^d^Adjusted odds ratio for gender, age

^e^Adjusted odds ratio for gender, age, education

^f^Adjusted odds ratio for gender, age, education, marital status

The likelihood ratios (LR) reveal that quality of life indicators and perceived health condition are potential PAL determinants in the entrepreneurs under study. The study models differed significantly (*p* < 0.001) from those composed of the intercept only. The comparison of LR models containing only quality of life and perceived health condition indicators (raw model—M_1_) with those adjusted for the variables of gender, age, education, and marital status (adjusted models—M_2_–M_5_) reveals a significant (*p* < 0.001) improvement in the goodness of fit of the analysed MLR models. The inclusion of confounding variables in the models also improved their predictive value. The accuracy levels (ACC) increased for models describing associations of PAL with OQoL and PHC by 12%; PAL with PHYD, PSYD, and SD by 6–10%; and PAL and ED by 15% (Table 4).

In the studied group of entrepreneurs, the odds of high rather than low PALs increased more than threefold (OR 3.16, CI 2.32–4.28) with higher overall quality of life scores. After adjusting for the confounding variables (gender, age, education, and marital status), the odds of a high rather than low PAL increased nearly fivefold (OR 4.86, CI 3.34–7.07) in those with higher quality of life scores. Also, the odds of moderate vs. low PAL increased with higher OQoL scores by nearly 30% regardless of the effect of confounding variables. However, this relationship was statistically non-significant (Table 4).

The entrepreneurs who rated their perceived health condition higher were almost twice as likely to report high rather than low levels of physical activity (OR 2.04, CI 1.57–2.63). Considering the respondents’ gender, age, education and marital status, the odds of HPAL versus LPAL were less determined by their health status (OR 1.92, CI 1.42–2.59). Moderate rather than low levels of physical activity were also more frequently found in the entrepreneurs who rated their perceived health condition higher. However, in this case, considering the combined effect of the independent variable of perceived health condition and CVs indicated the existence of a spurious relationship. The initial statistically significant relationship of PAL versus PHC (OR 1.32, CI 1.02–1.72) after accounting for gender, age, education, and marital status of entrepreneurs turned out to be statistically non-significant (OR 1.03, CI 0.77–1.38) (Table 4).

The conditional probability of high rather than low levels of physical activity tended to increase in the surveyed entrepreneurs with higher quality of life scores in the physical, psychological, social, and environmental domains by 50%, 20%, 23%, and 27%, respectively. The assessment of the combined effect of quality of life indicators in the above domains and of respondents' gender, age, education, and marital status showed an increased likelihood of HPAL rather than LPAL by 34% in the physical domain, 11% in the psychological domain, 5% in the social domain, and 6% in the environmental domain, respectively (Table 4).

The entrepreneurs who rated their quality of life in the physical domain higher also reported moderate rather than low levels of physical activity. This was found both when including (OR 1.54, CI 1.29–1.84) and not including (OR 1.37, CI 1.18–1.58) the confounding variables. The analysis of the relationship of MPAL with quality of life in the psychological, social, and environmental domains, showed that regardless of the effect of CVs, higher quality of life scores in these domains were accompanied by 3–10% lower odds of the MPAL rather than LPAL (statistically non-significant) (Table 4).

## Discussion

The World Health Organization Quality of Life (WHOQOL BREF) questionnaire was used as the main research tool in the present study focusing on health-related quality of life (HRQoL). Both the WHOQOL BREF and the HRQoL concept are of considerable methodological and practical significance, and their application situates the findings of the present study in line with those of other authors regarding the role of health-related quality of life in modeling the physical activity level of entrepreneurs.

The study results confirm the first research hypothesis. High levels of physical activity were reported by almost one half of the surveyed entrepreneurs from Wroclaw, and moderate levels of physical activity by more than a quarter of them. Also Gu et al. [24] in a study of a U.S. population representing various professions showed that white-collar workers were one of the most physically active groups as almost 60% of them met the guidelines of sufficient leisure-time physical activity. In the case of entrepreneurs, leisure-time physical activity appears to be of great importance since it is probably the prevailing component in the structure of their overall habitual physical activity. The nature of tasks performed by entrepreneurs and the length of their working time mean that physical exercise during their performance of professional duties, domestic chores, and commuting is usually limited. This was confirmed by studies on individuals of high socioeconomic status by Jurakic et al. [42]. The dominant role of leisure-time physical activity in the structure of overall physical activity of entrepreneurs is also evidenced by the negative correlations found by Kirk and Rhodes [43] between the level of physical activity and long work hours. Therefore, it is likely that the studied Wrocław entrepreneurs made rational use of their limited free time by performing various forms of physical activity. This is supported by their reported awareness of the significance of lifestyle for their health status (more than 80% of the entrepreneurs had a secondary or higher education). Other factors may have included affluence level, access to recreational infrastructure and services, and popularity of physical activity. Positive associations of physical activity with socioeconomic factors were empirically documented in earlier studies [44–46].

The second research hypothesis was also confirmed. A higher level of physical activity was reported by entrepreneurs with high perceived health condition. This is not surprising since good health is often a prerequisite for many forms of physical activity. It should be remembered, however, that perceived health condition does not solely depend on one’s objective health status, but is modified by psychological and cultural factors, socio-economic conditions, and lifestyle. For example, Trentini et al. [47] noted better perceived health condition in individuals who were well educated, belonged to the upper or upper middle class, and did not suffer from depression or low moods. Positive associations between physical activity levels and perceived health condition were found earlier by Cobley et al. [48], Greenley et al. [49], Koeneman et al. [50], and Olsen et al. [51].

The third research hypothesis was partially falsified. Among the entrepreneurs from Wrocław, positive associations of physical activity with overall quality of life and quality of life in the physical, psychological, and environmental domains were found. On the other hand, the correlations of physical activity with the quality of life in the social domain were bidirectional, i.e. higher physical activity levels were reported among entrepreneurs with better ratings of overall quality of life and quality of life in the physical and psychological domains. Entrepreneurs from Wrocław rated their quality of life in the physical domain (PHYD) on average 2.2 points lower (*p* < 0.001, d = 1.0—large effect) than those surveyed by Jaracz et al. [52] and 3.1–4.3 points lower (*p* < 0.001, d = 1.0–2.0—large effect) than respondents in Skevington et al. [53], Gholami et al. [54], Kalfoss et al. [55].

In the psychological domain (PSYD), the Wrocław entrepreneurs rated quality of life on average 0.6 points higher (*p* < 0.001, d = 0.3—small effect) than in the Jaracz et al. [52], study, 1.3 points lower (*p* < 0.001, d = 0.3—small effect) than in the Skevington et al. [53] study, and 2.2 points lower (*p* < 0.001, d = 1.1—large effect) compared to the observations of Kalfoss et al. [55].

In our study, quality of life in the social domain (SD) and environmental domain (ED) was found to be 0.5–0.9 points higher shaded (*p* < 0.001, d = 0.2–0.3—small effect) than in studies Jaracz et al. [52], Skevington et al. [53] i Gholami et al. [54]. The quality-of-life domains discussed were for SD by 0.4. points. (*p* < 0.05, d = 0.1—small to large effect), and ED by 2.7 pts. (*p* < 0.001, d = 1.2—large effect) lower rated by the surveyed entrepreneurs concerning the comparison study of Kalfoss et al. [55].

Siddiqi et al. [56] also observed positive correlations of physical activity with overall quality of life. Moreover, the results of many previous studies indicate a positive role of such aspects of quality of life in the physical domain as physical health [50], normal body weight [57, 58], stamina [51], or high physical capacity and fitness [59, 60] in modifying the physical activity level. It was also shown that experiencing pain, fatigue, and a sense of physical weakness can constitute a serious impediment to physical activity [56]. Researchers also stress the importance of psychological factors as potential determinants of physical activity levels. Stults-Kolehmainen and Sinha reported lower physical activity levels in people with high stress levels [61]. Self-efficacy and body weight control benefits were psychological modifiers of physical activity in Japanese female employees in Nishida et al. [62]. Also Caudroit et al. [63] described positive associations of physical activity with action and coping self-efficacy. Positive correlations between physical activity and physical self-concept and appearance were reported by Babic et al. [64] and Teixeira et al. [65]. Finally Rector et al. [66] proved that not only was the level of physical activity in adults positively correlated with their psychological well-being, but also that better well-being increased the likelihood of sustaining physical activity in the long term.

Positive relationships were also noted between physical activity and the environmental domain of quality of life among the Wrocław entrepreneurs. Correlations of physical activity with quality of life in the environmental domain were also reported by Garrett et al. [67], who concluded that good safety, accessibility of local resources, infrastructure, access to information, or material situation, and association with social factors are key in assessing the relationships between physical activity and quality of life in the environmental domain. Kamphuis et al. [68] found that favourable environmental conditions clearly influenced physical activity only in individuals from low socioeconomic groups. Jaime et al. [69], on the other hand, reported negative associations between physical activity and some environmental factors such as density of public transportation networks. Chen et al. [70] demonstrated negative correlations of occupational, transportation, and housework physical activity with financial situation, which is one of the components of quality of life in the environmental domain. On the other hand, Wendel-Vos et al. [71] reported no significant correlations of physical activity with environmental factors.

Correlations between physical activity and quality of life in the social domain were also found in the studied entrepreneurs from Wrocław: positive in respondents with high levels of physical activity, and negative in respondents with moderate physical activity levels. The results of previous studies, including our own, are not conclusive. Some authors failed to confirm any significant associations of physical activity with such components of quality of life in the social domain as community support or relationships with other people [72]. However, Anderson et al. [73] and Xiao et al. [37] demonstrated social support as a factor positively influencing the level of physical activity indirectly through its impact on self-efficacy.

The present study also revealed a modifying effect of gender, age, education and marital status on the relationships between physical activity and quality of health-related quality of life. The impact of confounding variables was particularly evident in the relationship between physical activity and quality of life in the physical and social domains and perceived health condition.

The present study has its strengths and weaknesses. The strengths include the research material and issues since the relationships between physical activity and quality of life among entrepreneurs have not been considered before. Also, health-related quality of life has not been analyzed previously as a determinant of physical activity among entrepreneurs. This is because earlier studies only investigated the potential impact of physical activity levels on health-related quality of life. The weaknesses of the present study include its cross-sectional nature, spatial scope limited to one city, and the fact that the surveyed entrepreneurs were examined together. In the context of these findings, it is therefore imperative to continue studies on physical activity and quality of life of entrepreneurs. Further research should be conducted in the form of prospective cohort studies with a wider spatial scope and cover the whole of Poland or other Central and Eastern European countries. Quality of life concepts other than health-related quality of life, for example, economic, environmental and socio-psychological should also be considered. Analysis of relationships between physical activity and quality of life in separate groups based on gender, age, level of education and type, form and size of business activity is also worth considering. It may also be interesting to carry out a separate analysis of relationships between quality of life and physical activity in terms of domains in which it is undertaken, e.g. leisure-time PA, occupational PA, active transportation PA, and different physical activity forms, e.g. running, cycling, swimming, etc.

## Conclusions

The study results confirmed the first research hypothesis that the majority of the surveyed entrepreneurs in Wrocław were characterized by a high level of physical activity. The second hypothesis should also be fully accepted since the entrepreneurs’ level of physical activity turned out to be positively related with perceived health condition. However, the third research hypothesis is partially falsified. The level of physical activity is positively related to the overall quality of life and quality of life in the physical, psychological and environmental domains. On the other hand, the associations of physical activity with the quality of life in the social domain were bidirectional: positive for a high level of activity and negative for a moderate level of physical activity. However, it should be emphasized that these findings refer only to health-related quality of life in a population of entrepreneurs.

In the context of these findings, it is desirable to implement comprehensive programs to stimulate physical activity in the workplace, while commuting, and in leisure time. These programs should include, first of all, the possibility for entrepreneurs to undertake physical activities of moderate and vigorous intensity. Activities aimed at improving health-related quality of life in the physical, psychological, social and environmental domains as a potential determinant of physical activity levels are also worth recommending. The relationships between physical activity and health-related quality of life also indicate the need to continue the implementation of the concept of wellbeing in modern business companies. Taking care of the physical, mental, intellectual, and spiritual wellbeing of employees and entrepreneurs themselves, including favourable relations with the surrounding environment, will increase commitment, satisfaction and productivity, reduce absenteeism, and—consequently—improve the image and quality of business enterprises. It can also serve as a basis for the application of other theoretical concepts and tools measuring quality of life in future research, and for making further comparative analysis and recommendations.

## Data Availability

The datasets used and/or analyzed in the study are available from the corresponding author on reasonable request.
